# Expression of major photosynthetic and salt-resistance genes in invasive reed lineages grown under elevated CO_2_ and temperature

**DOI:** 10.1002/ece3.1282

**Published:** 2014-10-12

**Authors:** Franziska Eller, Carla Lambertini, Mette W Nielsen, Simona Radutoiu, Hans Brix

**Affiliations:** 1 Department of Bioscience, Aarhus UniversityOle Worms Alle 1, Aarhus C, DK-8000, Denmark; 2 Biocenter Klein Flottbek, Hamburg UniversityOhnhorststrasse 18, Hamburg, D-22609, Germany; 3 Department of Molecular Biology and Genetics, Aarhus UniversityGustav Wieds Vej 10, Aarhus C, DK-8000, Denmark

**Keywords:** Common reed, Delta-type, EU-type, Mississippi River Delta, *Phragmites australis*, US Gulf Coast

## Abstract

It is important to investigate the molecular causes of the variation in ecologically important traits to fully understand phenotypic responses to climate change. In the Mississippi River Delta, two distinct, sympatric invasive lineages of common reed (*Phragmites australis*) are known to differ in several ecophysiological characteristics and are expected to become more salt resistant due to increasing atmospheric CO_2_ and temperature. We investigated whether different patterns of gene expression can explain their ecophysiological differences and increased vigor under future climatic conditions. We compared the transcript abundance of photosynthetic genes of the Calvin cycle (Rubisco small subunit, *RbcS*; Phosphoglycerate kinase, *PGK*; Phosphoribulokinase, *PRK*), genes related with salt transport (Na^+^/H^+^ antiporter, *PhaNHA)* and oxidative stress response genes (Manganese Superoxide dismutase*, MnSOD*; Glutathione peroxidase, *GPX*), and the total aboveground biomass production between two genotypes representing the two lineages. The two genotypes (Delta-type, Mediterranean lineage, and EU-type, Eurasian lineage) were grown under an ambient and a future climate scenario with simultaneously elevated CO_2_ and temperature, and under two different soil salinities (0‰ or 20‰). We found neither differences in the aboveground biomass production nor the transcript abundances of the two genotypes, but soil salinity significantly affected all the investigated parameters, often interacting with the climatic conditions. At 20‰ salinity, most genes were higher expressed in the future than in the ambient climatic conditions. Higher transcription of the genes suggests higher abundance of the protein they code for, and consequently increased photosynthate production, improved stress responses, and salt exclusion. Therefore, the higher expression of these genes most likely contributed to the significantly ameliorated salinity impact on the aboveground biomass production of both *P. australis* genotypes under elevated temperature and CO_2_. Although transcript abundances did not explain differences between the lineages, they correlated with the increased vigor of both lineages under anticipated future climatic conditions.

## Introduction

Greenhouse gas concentrations in the atmosphere are rising significantly due to anthropogenic fuel burning, with concomitant increases in average global temperature (IPCC 2007; Meehl et al. 2012). The atmospheric CO_2_ concentration, one of the major greenhouse gases, has been predicted to reach 700 ppm by the end of the 21st century, double that of just two centuries ago (Barnola et al. 1995; IPCC 2007). Among the consequences of global warming are recurring storm events, the thermal expansion of the oceans and the melting of the polar ice caps (IPCC 2007). The resulting seawater expansion into coastal areas is a threat to coastal ecosystems, especially the fresher, upper marsh vegetation.

Elevated concentrations of atmospheric CO_2_ can mitigate salt stress in plants (Poorter and Perez-Soba 2001), as leaf transpiration is decreased at high CO_2_ levels, due to lower stomata conductance. As a result, plant water status can be improved. Moreover, in C_3_ plants, elevated CO_2_ often results in higher photosynthesis rates, which can lead to improved osmotic adjustment (Perez-Lopez et al. 2009a). The surplus of C-substrates supplied by elevated CO_2_ can also increase the synthesis and activity of enzymes involved in the detoxification of reactive oxygen species (ROS), and thus, reduce salinity damages caused by ROS (Perez-Lopez et al. 2009b; Geissler et al. 2010). As photosynthetic stimulation by elevated CO_2_ has been shown to be greater under higher temperature regimes (Ainsworth and Long 2004), future climatic conditions may be highly beneficial for plant resistance to salinity.

The common reed (*Phragmites australis* (Cav.) Trin. ex Steud.) is a robust perennial wetland grass with a broad ecological amplitude, as evidenced by its cosmopolitan distribution and diversity of habitats, ranging from fresh to brackish and even saline waters (Haslam 1972; Brix 1999; Chambers et al. 2003). Large phenotypical, morphological, anatomical, and ecophysiological differences between and within the distinct reed genotypes and lineages have previously been reported (Clevering et al. 2001; Lessmann et al. 2001; Bastlova et al. 2006; Hansen et al. 2007; Eller and Brix 2012; Eller et al. 2013, 2014; Nguyen et al. 2013).

**Figure 1:**
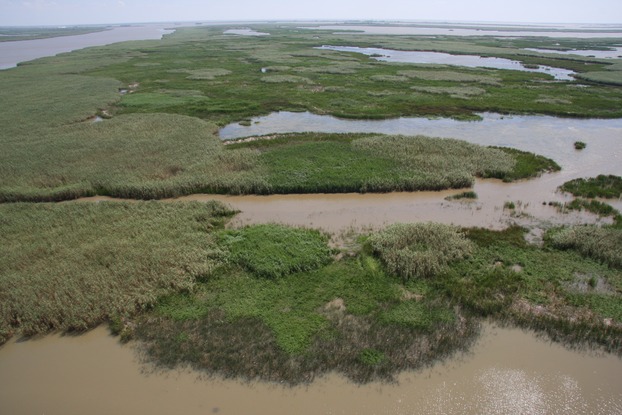
Phenotypically distinguishable, monoclonal patches of different invasive *Phragmites australis* types in the Mississippi River Delta. Photo: H. Brix.

*Phragmites australis* is a natural component of North American wetlands (Orson 1999), but over the last century a Eurasian lineage has been introduced and is spreading rapidly along the North American Atlantic coast (Saltonstall 2002; Meyerson et al. 2010). Differences in salt tolerance between the native and the Eurasian lineage may have allowed the colonization of habitats unavailable to the native lineage (Vasquez et al. 2005). In the Mississippi River Delta, many more exotic genotypes of different *P. australis* lineages have been spreading (Hauber et al. 2011; Lambertini et al. 2012a). These phenotypically distinguishable reeds occur within the Delta mostly in adjacent monoclonal patches, and hence, with limited genotypic variation within a patch (Fig. 1). Among these, a lineage originating in the arid Mediterranean regions of Europe, North Africa and the Middle East (Lambertini et al. 2012b), appears to be coping better with the brackish conditions of the Delta, given its dominance in the area over the other *Phragmites* lineages and its predominant occurrence in the outer marshes of the Delta. The native *P. australis* ssp. *americanus* is largely absent in the Mississippi River Delta, and the aggressively spreading Eurasian lineage has only recently been introduced to this area where it is competing mainly with the Mediterranean lineage (White et al. 2004; Hauber et al. 2011; Lambertini et al. 2012a).

These two exotic lineages do not only differ in the distribution in their native ranges, but also in their morphology and physiology (Hauber et al. 2011; Lambertini et al. 2012a,b; Guo et al. 2013; Nguyen et al. 2013). We have previously shown that a genotype of the Mediterranean lineage had superior photosynthetic traits and higher salt resistance than a genotype of the Eurasian lineage (Eller et al. 2014). In that study, we also found that simulated future climatic conditions with elevated CO_2_ and temperature favoured growth, photosynthesis, water use-efficiency and several other ecophysiological traits of these two competing exotic reed lineages. Overall, these observations have shown that both invasive lineages will benefit from future climatic conditions and that their invasive vigor will be enhanced.

However, to fully understand the phenotypic responses to climate change, it is important also to investigate the molecular causes of the variation in ecologically important traits. Differences in gene expression between the two exotic reed lineages are likely to cause, or underlie, the different phenotypic traits. Differences in salt tolerance between distinct *P. australis* genotypes have previously been ascribed to the differential expression of a potassium transporter gene (Takahashi et al. 2007), which shows how distinct physiological reed phenotypes can be explained by molecular studies. The transcript abundance is to a certain degree correlated with the abundance of the respective protein, and the abundance of an enzyme implies the level of its catalytic activity (Baerenfaller et al. 2008; Maier et al. 2009; Vogel and Marcotte 2012). We would thus expect the alleviated salinity impact and the higher photosynthetic rates of the genotypes under a future climate scenario to be caused by increased expression of the involved genes.

In this study, we compared the transcript abundance of genes involved in photosynthesis, salt transport, and oxidative stress response between two salt-exposed genotypes of the two invasive lineages (represented by the Delta-type, Mediterranean lineage and the EU-type, Eurasian lineage) under an ambient and a future climate scenario with simultaneously elevated CO_2_ and temperature. Previous studies (Lambertini et al. 2012a; Nguyen et al. 2013; Eller et al. 2014) showed clearly that these two genotypes are highly representative of their respective lineage, and that any variation within lineages was considerably smaller than between. In general, multifactorial effects on gene expression are rarely investigated but can elucidate how plants are responding to naturally interacting factors on the molecular level. We first hypothesized that a higher expression of the photosynthetic and salinity-related genes is responsible for the previously observed higher photosynthetic capacity and salt tolerance of the Mediterranean compared with the Eurasian genotype. Secondly, we hypothesized that under elevated CO_2_ and temperature, the expression of salinity-related genes would be increased in both invasive lineages due to the expected ameliorated salinity impact in the future climate scenario.

## Materials and Methods

### Plant material

Rhizomes of *Phragmites australis* with EU and Delta phenotypes (sensu Hauber et al. 2011; Lambertini et al. 2012a) were collected in the Mississippi River Delta in June 2009 and grown in a common environment at Aarhus University, Denmark (56°13′N; 10°07′E), for at least one year prior to the study. The two phenotypes were genotyped with chloroplast and nuclear DNA as haplotype M, Eurasian lineage, and haplotype M1, Mediterranean lineage, respectively (Lambertini et al. 2012a). One genotype that had previously been found to be a good representative of each lineage was chosen for this experiment: ROM2 (Eurasian lineage, hereafter called EU-type) and ROMS4 (Mediterranean lineage, hereafter called Delta-type), following the nomenclature in Nguyen et al. (2013).

Shoots were layered horizontally in water for five weeks to initiate adventitious shoot growth at the stem nodes, to produce replicates of genetically identical shoots of the two genotypes. When the adventitious shoots had developed roots and had reached a height of approximately 0.15–0.20 m, the stems were cut at both sides of the nodes, and the resulting replicate plants were planted in 3.5 L pots with commercial peat. The pots were placed in rectangular plastic containers (0.55 m l × 0.12 m h × 0.36 m d) with tap water to ensure well-watered conditions throughout the acclimation period, on tables in two walk-in growth chambers in the climate phytotron ʻRisø Environmental Risk Assessment Facilityʼ (RERAF, DTU, Denmark). The tables and pots were rotated within the chambers on a weekly basis to counteract chamber and edge effects. All plants were fertilized weekly with 0.5 L of a nutrient solution containing a commercial fertilizer (Substral®, The Scotts Company, Nordics A/S, Glostrup, Denmark). Extra micronutrients were supplied from a micronutrient stock solution (Pioner Mikro Plus Fe, Brøste, Lyngby, Denmark). Additional iron was added as Fe(II)SO_4_ to the nutrient solution immediately before every fertilization, to avoid the often occurring chlorosis in young *P. australis* leaves.

### Climatic treatment

The plants were grown at either (1) a CO_2_ concentration of 390 ppm and a 19/12 °C day/night temperature regime (ambient climatic conditions) or at (2) a CO_2_ concentration of 700 ppm and a 24/17 °C day/night temperature regime (elevated climatic conditions) in two physically and electronically separated, gas tight walk-in growth chambers (6 m w, 4 m l, 3.1 m h). The treatment conditions in the elevated climatic conditions were chosen to conform to the forecast global atmospheric CO_2_ and temperature changes by the end of the 21st century (IPCC 2007; Meehl et al. 2012). We focused on the combined effect of CO_2_ and temperature only, as the effects of CO_2_ and temperature as single factors on *P. australis* vary considerably from their combined effect (Eller et al. 2013). Hence, the “ambient climatic conditions” and “elevated climatic conditions” were considered as single treatments. The chosen temperature regimes resembled cooler seasons which the reeds also face at the early onset of the growing season in the Mississippi River Delta region. Under these relatively mild temperature regimes, we assured that photosynthesis was limited by Rubisco activity and not by a decreased activation state of Rubisco due to too hot conditions, because heat stress was not the focus of this study.

Each chamber was equipped with independent ventilation systems and individually controlled light, temperature, humidity, and CO_2_. The air within each chamber was mixed by two opposite fans. High pressure mercury and halogen lamps supplemented the natural sunlight entering through the transparent roof, providing an irradiance of approximately 400 *μ*mol/m^2^/s (photosynthetic photon flux density, PPFD) at shoot base. The day/night regime was 12/12 h. During the first and last hour of the day, sunrise and sunset were simulated by a gradually changing light intensity. The relative air humidity was set to be 55/70% (day/night). The monitored environmental conditions were stable throughout the experimental period (data not shown).

### Salinity treatment

After one week of acclimation in the growth chambers, half of the plants (*n* = 8) in each climate treatment were exposed to increased soil water salinity. The pots of the salt-treated plants were thoroughly flushed with a 10‰ salt solution prepared from a commercial salt (98.5% NaCl) in tap water. The outer plastic containers holding the salt-treated plants were then filled to 7 cm height with the salt solution. After 1 week, salinity was increased to 20‰ and the pots thoroughly flushed, first with tap water to leach the previous salts from the soil, and then with the saline solution. Control plants (0‰ salinity) were flushed with tap water instead of saltwater but otherwise treated in a similar way as the salt-treated plants. The 20‰ salinity level was chosen based on the current understanding of the salt tolerance of *P. australis* (Lissner and Schierup 1997; Moore et al. 2012) to make sure that the plants were affected, but not killed. The flushing of the pots was repeated every 7 days to ensure a close to constant salt concentration in the pots, and the solution in the plastic containers holding the pots was completely replaced. Between the flushing, the plants were watered with tap water to replace water lost by evapotranspiration.

### Experimental set-up

The experimental setup was a 2 × 2 × 2 factorial design with the factors genotype (“EU-type” vs. “Delta-type”), climatic conditions (“ambient” vs. “elevated”), and soil salinity (“0‰ salinity” vs. “20‰ salinity”) with eight replicates in each treatment combination. Only three of the replicates were used for gene expression analysis, but all were used to determine the aboveground biomass.

### RNA extraction and quantification

After 66 days of growth under the treatment conditions, three to four young, fully developed leaves (taken from two to three shoots of similar age) per plant of three replicates within each treatment were harvested and immediately frozen in liquid nitrogen. The leaves were then pulverized in liquid nitrogen using a porcelain mortar and pestle. RNA of each replicate was extracted from the leaf material using the NucleoSpin® RNAII Kit (Macherey-Nagel, Düren, Germany) following the manufacturer′s instructions. The purity of the total RNA extracted was determined as the 260/280 nm and 260/230 nm absorbance ratio, and the integrity of the RNA was checked by electrophoresis in a 0.8% agarose gel.

### Reverse transcription

The cDNA was reverse transcribed using the First-Strand cDNA Synthesis Kit, including DNase treatment (GE Healthcare Life Sciences Pittsburgh, PA, USA), from 4 *μ*g of total RNA following the manufacturer's instructions.

### Genes studied and primer design

The genes selected for this study are nuclear genes involved in photosynthesis, salt resistance and oxidative stress response. The photosynthesis-related genes code for the small subunit of Rubisco (*RbcS*), Phosphoglycerate kinase (*PGK*), and Phosphoribulokinase (*PRK*), which are enzymes involved in the Calvin Cycle. The salt resistance-related genes code for the Na^+^/H^+^ antiporter (*PhaNHA*), which is involved in Na^+^ transport, and for Manganese Superoxide dismutase (*MnSOD*) and Glutathione peroxidase (*GPX*), which are ROS detoxifying enzymes. The gene coding for Ubiquitin-conjugating enzyme (*UBC*) is a housekeeping gene, and this gene was used as reference of stable gene expression throughout the different treatments. Gene sequences were obtained from sequences in Genbank of *P. australis* (Davies et al. 2009; Takahashi et al. 2009), and from other species of the Poaceae family (Smith et al. 1983; White and Scandalios 1988; Longstaff et al. 1989; Raines et al. 1989; Churin et al. 1999; Chen et al. 2004; Sreenivasulu et al. 2004; Kumari et al. 2009; Wu et al. 2011). The gene sequences were aligned with the sequence alignment software MultAlin (Corpet 1988), and forward (fwd) and reverse (rev) primers were designed in the regions that scored the highest homology among alignments of up to 12 different Poaceae genera (*Oryza*, *Zea*, *Hordeum*, *Triticum*, *Secale*, *Setaria*, *Sorghum*, *Bambusa*, *Lolium*, *Phyllostachys*, *Agropyron*, *Puccinellia*). The primers were located to include an intron, to avoid the amplification of possible genomic DNA in the cDNA. The targeted gene regions were sequenced both from the cDNA and the genomic DNA. Primers used for real-time quantitative PCR (RT-qPCR) are listed in Table 2. The identity of the RT-qPCR products was confirmed by Sanger sequencing in an ABI sequencer (Applied Biosystems, Foster City, CA). The resulting sequences were edited with the sequence alignment program BioEdit v7.0.9 (Hall 1999).

### RT-qPCR and gene expression data analysis

Real-time quantitative PCR (RT-qPCR) with the target and reference gene-specific primers was performed with a LightCycler® 480 (Roche Molecular Diagnostics Pleasanton, CA, USA) using LightCycler 480 SYBR Green I Master (Roche Molecular Biochemicals) and 1 *μ*L of 25-fold diluted cDNA template for each RT-qPCR reaction. Each 20 *μ*L reaction mixture contained 10 *μ*L of 2 × SYBR Green I Master mix, 1 *μ*L of each primer (10 *μ*mol/L), 1 *μ*L of sample cDNA, and 7 *μ*L of sterilized ultra-pure H_2_O. PCR conditions were denaturation at 95 °C for 10 min, the respective annealing temperature for the primer pair for 5 s (Table 1), followed by 55 elongation cycles at 72 °C for 10 s, and the respective melting temperature for the primer pair for 1 s (Table 1). No-template controls were included in each run using sterilized ultra-pure H_2_O instead of template cDNA. Each replicate sample was run three times in the RT-qPCR (technical replicates).

**Table 1:** Primers designed for real-time quantitative PCR of genes of invasive *Phragmites australis*.

Gene	Primer	Primer sequence	*T*_a_ (°C)	*T*_m_ (°C)
*RbcS*	rbcS-fwd	5′ GAT CAG GTG CAT GCA GGT GTG G 3′	56	84
	rbcS-rev	5′ CCG ACC TTG CTG AAC TCG AGG 3′		
*PGK*	Phgly-fwd	5′ GTT TGC TGT AGG AAC TGA GGC TGT 3′	57	82
	Phgly-rev	5′ CAC CTC CCG TTG AAA TGT GGC TCA 3′		
*PRK*	Phori-fwd	5′ GAC TCT TAC TTC GGC CAT GAG GTA TCA G 3′	55	80
	Phori-rev	5′ GAA GAG ACC TGT TCC ATT GTT GCT 3′		
*PhaNHA*	NaH-fwd	5′ GTG CGG CTT TTG AAT GGT GTG CA 3′	55	79
	NaH-rev	5′ GGG AAC TGG ACA CTG GAC TGT AAA 3′		
*GPX*	GPX-fwd	5′ GAA TTC CCT ATT TTT GAC AAG GTT GA 3′	55	77
	GPX-rev	5′ GCG CAT AGC GAT CCA CAA C 3′		
*MnSOD*	SOD-fwd	5′ CAA GGA TCT GGA TGG GTG TGG C 3′	55	80
	SOD-rev	5′ GTA GTA CGC ATG CTC CCA GAC AT 3′		
*UBC*	UBC-fwd	5′ CTT CAA GCC RCC AAA GGT MTC 3′	55	80
	UBC-rev	5′ GAT ATT GTC AAA GCA GGG CTC CA 3′		

*T*_a_, annealing temperature of primer pair; *T*_m_, melting temperature of primer pair.

Melting curves were run to identify the crossing point (*C*_p_) of all samples. To ensure an equal concentration of cDNA, all samples were diluted to reach the same *C*_p_ (±1 cycle). A standard serial dilution series of a mixture of cDNA from all samples of the analysis (called calibrator) was made to correct for the PCR efficiency. To determine the relative transcript abundance of each gene, the quantification software (Roche Molecular Biochemicals) was used, which incorporated the correction for PCR efficiency, and *C*_p_ values for normalization of the target to the reference gene UBC.

The geometric means (Vandesompele et al. 2002) of the relative expression ratios for the three biological and three technical replicates were calculated. Data were transformed to the natural logarithm for analysis with Statgraphics Centurion XVI (Statpoint Technologies, Inc., Warrenton VA, USA) and tested for variance homogeneity using Bartlett's test. The General Linear Models (GLM) procedure was used with the factors “genotype”, “climatic conditions” and “soil salinity” and their interactions (*n* = 3). To detect differences due to treatment effects, post hoc comparisons of means at the 95% confidence level were made by Tukey's honestly significant differences (HSD) procedure. As no significant effect or interaction was found for the factor “genotype”, data for both genotypes were subsequently pooled and analyzed by a two-way Analysis of Variance (ANOVA) using ln-transformed data, to specify and simplify the analysis. The variance homogeneity was tested using Bartlett's test. Tukey's HSD tests were made post hoc to detect differences between the means. The untransformed data are shown as expression relative to the ambient climatic conditions and 0‰ salinity, which was chosen to show a relative expression of 100.

### Aboveground biomass analysis

After 70 days of growth, the aboveground plant parts of all replicates (*n* = 8 for each treatment and genotype) were harvested and dried to constant weight at 80°C in a ventilated oven to determine their final biomass. Aboveground biomass data were analyzed by GLM as described for the gene expression data. Also for the aboveground biomass, no significant effect or interaction was found for “genotype”. Hence, data for both genotypes were pooled, and the log-transformed data were analyzed by a two-way Analysis of Variance (ANOVA) (*n* = 8) as described above.

## Results

### RNA purity and integrity

The RNA obtained from the *P. australis* leaves was of good quality and suitable for RT-qPCR, as suggested by Udvardi et al. (2008). The average concentration of RNA extracted was 238 ng RNA_/_*μ*l. The average A_260_/A_280_ ratio of 2.1 indicated that there was no protein contamination in the preparation. Also, the agarose gels proved high integrity of the isolated RNA (not shown).

### Gene sequences

All sequences obtained from the RT-qPCRs could be aligned with the respective mRNA sequences of other Poaceae species in Genbank. The accession numbers and gene product function for the *P. australis* partial gene sequences obtained are summarized in Table 2. There were no differences in the cDNA sequences between the EU-type and the Delta-type.

**Table 2:** Genes of invasive *Phragmites australis* analyzed by real-time quantitative PCR. Plants were grown under ambient or elevated climatic conditions, at 0‰ or 20‰ salinity, for 70 days before harvesting.

Gene	Name	Function	Type	Accession numbers
*RbcS*	Rubisco small subunit	CO_2_ fixation in 3-Phosphoglycerate	Genomic DNA; EU-type	KC130867
			Genomic DNA; Delta-type	KC130866
			mRNA	KC130859
*PGK*	Phosphoglycerate kinase	Phosphorylation of 3-Phosphoglycerate	Genomic DNA	KC130864
			mRNA	KC130856
*PRK*	Phosphoribulokinase	Phosphorylation of Ribulose-5-phosphate, regenerating Rubisco substrate RuBP	Genomic DNA	KC130865
			mRNA	KC130857
*PhaNHA*	Na^+^/H^+^ antiporter	Membrane protein, Na^+^ translocation	Genomic DNA	KC130863
			mRNA	KC130855
*MnSOD*	Mn^2+^ Superoxide dismutase	Dismutation of superoxide into O_2_ and H_2_O_2_	Genomic DNA	KC130862
			mRNA	KC130854
*GPX*	Glutathione peroxidase	Reduction of H_2_O_2_ to H_2_O	mRNA	KC130853

### Expression of photosynthesis-related genes

Ubiquitin-conjugating enzyme (UBC) was stably expressed in all treatments and hence served as reliable reference gene (data not shown). The GLM revealed that the transcript abundance of the three photosynthesis-related genes was not statistically different between the two genotypes (Tables S1 and S2). As a result, the genotypes were pooled in the further statistical analysis. The two-way ANOVA showed that “climatic conditions” significantly affected the expression of *PRK,* while “soil salinity” affected the expression of all photosynthesis-related genes. However, the effects of soil salinity on the expressions of *RbcS* and *PRK* depended on the climatic treatment, as indicated by the significant interaction terms in the ANOVA (Table 3).

**Table 3:** *F*-ratios of two-way Analysis of Variance (ANOVA) of aboveground biomass and transcript abundance of genes expressed in two invasive genotypes of *Phragmites australis* from the Mississippi River Delta. Data from the two genotypes did not differ statistically significantly for any of the parameters; hence, data are pooled in this analysis. The main factors are “climatic conditions” (Clim; ambient climatic conditions vs. elevated climatic conditions) and “soil salinity” (Sal; 0‰ salinity vs. 20‰ salinity).

Parameter	Main factors		Interactions
	
	Clim (df = 1)	Sal (df = 1)	Clim × Sal (df = 1)
*RbcS*	0.40	6.63*	12.94**
*PGK*	3.50	4.42*	4.04
*PRK*	4.80*	9.70**	4.51*
*PhaNHA*	0.92	6.82*	2.03
*MnSOD*	1.14	20.50***	4.40*
*GPX*	0.32	21.61***	45.53***
Aboveground biomass	405.78***	565.51***	0.10

RbcS, Rubisco small subunit; PGK, Phosphoglycerate kinase; PRK, Phosphoribulokinase; PhaNHA, *P. australis* Na^+^/H^+^ antiporter; MnSOD, Manganese Superoxide dismutase; GPX, Glutathione peroxidase; df, degrees of freedom.

Statistically significant values are shown in bold: *<0.05 probability level, **<0.01, ***<0.001.

**Figure 2:**
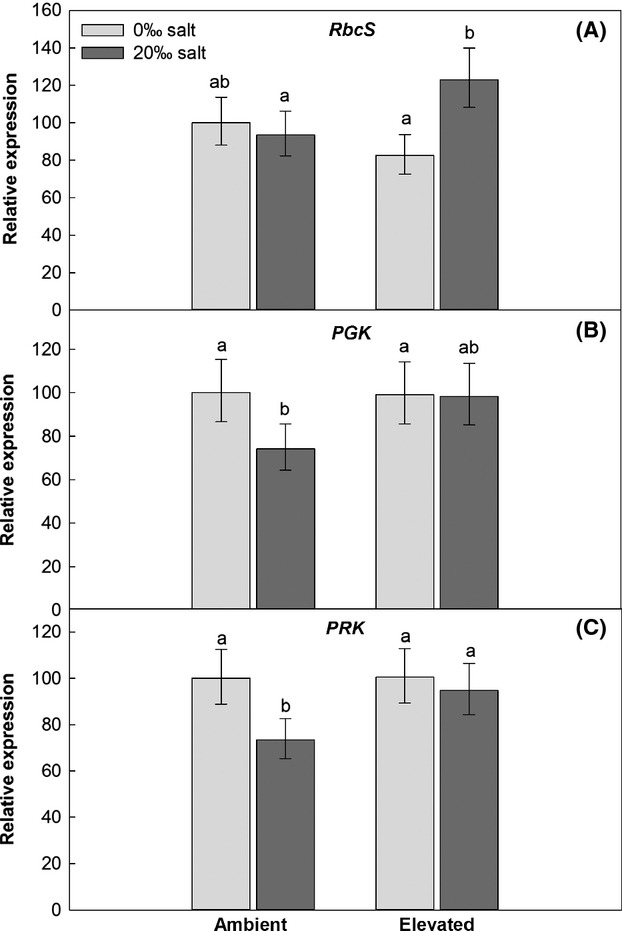
Relative expression of photosynthesis-related genes, pooled averages of two genotypes belonging to two invasive *Phragmites australis* lineages, grown under “ambient” and “elevated” climatic conditions, and at 0‰ or 20‰ salinity. Error bars show Tukey's HSD intervals, letters indicate statistically significant differences. Rubisco small subunit (*RbcS*) (A) Phosphoglycerate kinase (*PGK*) (B), Phosphoribulokinase (*PRK*) (C).

The expression of *RbcS* was not significantly affected by soil salinity under the ambient climatic conditions. Under the elevated climatic conditions, however, *RbcS* transcript abundance was 49% higher at 20‰ compared to 0‰ salinity (Fig. 2A). The 20‰ salinity decreased the transcript abundance of *PGK* by 26% under the ambient climatic conditions, but only by ∼1% under the elevated climatic conditions (Fig. 2B). Also, the expression of *PRK* was decreased at 20‰ salinity, but only under the ambient climatic conditions, where the transcript abundance was about 27% lower compared to 0‰ salinity (Fig. 2C).

### Expression of salt resistance-related genes

The GLM showed that the transcript abundances of the two genotypes did not differ for any of the three salt resistance-related genes (Tables S1 and S2). The two-way ANOVA of the pooled data showed that “soil salinity” significantly affected the expression of all three salt resistance-related genes, and that *MnSOD* and *GPX* expression were affected by a “climatic conditions × soil salinity” interaction (Table 3).

**Figure 3:**
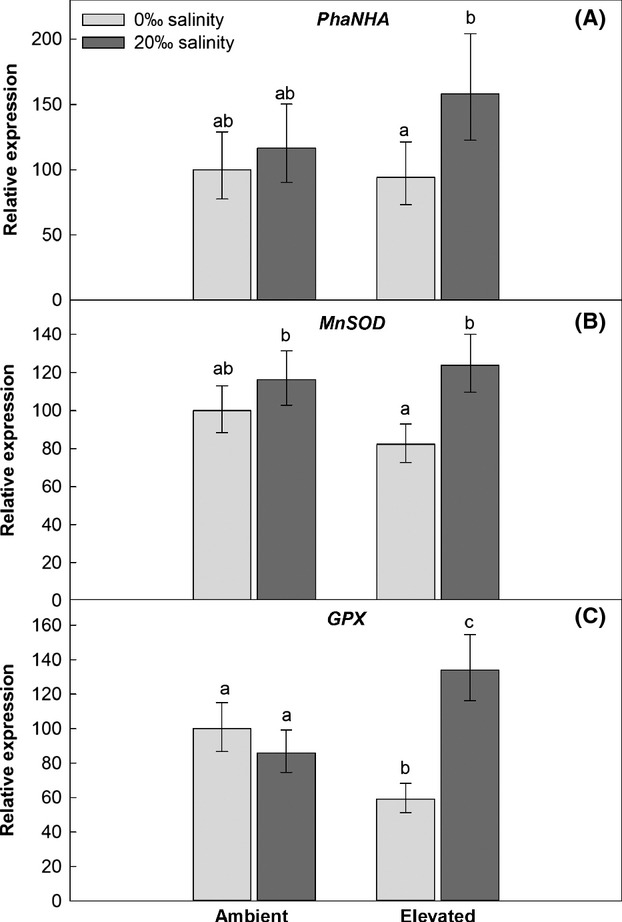
Relative expression of salt resistance-related genes, pooled averages of two genotypes belonging to two invasive *Phragmites australis* lineages grown under “ambient” and “elevated” climatic conditions, and at 0‰ or 20‰ salinity. Error bars show Tukey's HSD intervals, letters indicate statistically significant differences. Na^+^/H^+^ antiporter (*PhaNHA*) (A), Manganese Superoxide dismutase (*MnSOD*) (B), Glutathione peroxidase (*GPX*) (C).

The *PhaNHA* transcript abundance was higher in the salt-treated plants compared with nonsalt-treated plants, in the ambient climatic conditions about 16%, and in the elevated climatic conditions about 68% (Fig. 3A).

Under the ambient climatic conditions, the transcript abundance of *MnSOD* was ∼16% higher at 20‰ salinity compared to 0‰ salinity. However, the difference was more pronounced under the elevated climatic conditions, with 51% higher expression in the salt-treated plants (Fig. 3B).

In plants without salt treatment, *GPX* expression was 41% lower at elevated than at ambient climatic conditions. In the salt-treated plants, the transcript abundance was 56% higher at elevated compared with ambient climatic conditions (Fig. 3C).

### Total aboveground biomass

**Figure 4:**
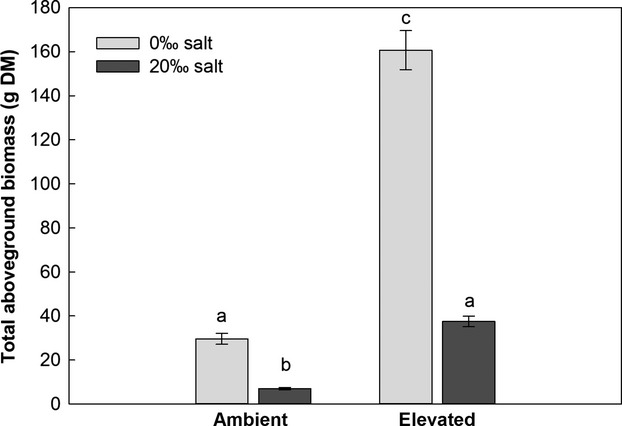
Total aboveground biomass (g dry mass, DM; *n* = 8; mean ± 1 SE), pooled averages of two genotypes belonging to two invasive *Phragmites australis* lineages, grown under “ambient” and “elevated” climatic conditions, and at 0‰ or 20‰ salinity. Letters indicate statistically significant differences.

The GLM showed that there was no significant difference between the aboveground biomass production of the two genotypes (Tables S1 and S2). The two-way ANOVA of the pooled data showed that “soil salinity” and “climatic conditions” significantly affected the aboveground biomass (Table 3). The biomass production was about five times higher at elevated compared to ambient climatic conditions, mainly due to the large production of runners, also called “legehalme”. Salinity negatively affected biomass development in both climatic treatments, and the salt-treated plants had about four times less biomass than the plants grown at 0‰ salinity (Fig. 4).

## Discussion

The aim of our study was to investigate the effect of climatic conditions on the gene expression of two genotypes, representing two invasive *Phragmites australis* lineages from the Mississippi River Delta, to detect possible differences between the genotypes. The two lineages, the Eurasian haplotype M and the Mediterranean haplotype M1, were previously shown to differ significantly in their salt resistance and ecophysiology (Nguyen et al. 2013; Eller et al. 2014). We hypothesized that genotypic differences in the transcript abundance of genes related to salt resistance and photosynthesis would be the underlying reason for this observation, assuming that the mRNA concentration is a proxy for the concentration and activity of the corresponding proteins (de Sousa Abreu et al. 2009). However, we could not detect any differences in the expression of the investigated genes between the two genotypes. Our two climatic treatments were less hot and humid than the conditions in the Mississippi River Delta. However, according to Guo et al. (2013), both lineages have a wide environmental tolerance and can occupy a broader climatic niche in North America than anticipated so far, also including areas with temperate climatic regimes as used in this study. Moreover, the two genotypes have previously been shown to differ in several photosynthetic traits under the same conditions as used here (Eller et al. 2014). This suggests that the chosen climatic conditions cannot account for our inability to confirm our first hypothesis. It is more likely that other factors were responsible for the phenotypic dissimilarities of the plants, such as other genes than those investigated here, epigenetic influences, or differences in protein turnover.

Although changes in gene expression are rapid and can even be more pronounced than changes in protein abundance and metabolic activity (Osuna et al. 2007), the correlation between the amount of mRNA and the gene product may be weak in certain organisms due to post-transcriptional modifications and other regulatory processes (Vogel and Marcotte 2012). Also, local gradients of transcriptional differences within plant tissue have previously been observed (Li et al. 2010) and could explain why we did not detect any differences between the two genotypes, as we analyzed pooled leaf material.

Despite the similar transcript abundances of the two genotypes, the expression of all the genes investigated was significantly affected by at least one of the two treatment factors. The *RbcS, PGK,* and *PRK* transcripts of the two *P. australis* genotypes were similar in the ambient and the elevated climatic conditions at 0‰ salinity. Like RbcS, PGK is involved in carbon fixation, following Rubisco as the next enzymatic step in the Calvin Cycle. PRK is related to the regeneration of the Rubisco substrate and catalyses the last enzymatic step of the Calvin Cycle. A common response of C_3_ plants grown at elevated CO_2_ is photosynthetic acclimation, implemented by downregulation of the *RbcS* transcript abundance, compared with the *RbcS* transcript abundance of plants grown at ambient CO_2_ (Paul and Driscoll 1997; Gesch et al. 2003). The Rubisco protein content and activity decrease correspondingly, although not to the same extent as the gene expression (Gesch et al. 2003). Our results indicate that the two genotypes showed no photosynthetic acclimation under the elevated climatic conditions, as the photosynthetic genes were not downregulated. Our elevated climatic treatment also included elevated temperature, which may have affected the transcription level and plant development (Taylor et al. 2005; Ainsworth et al. 2006; Li et al. 2008). This would explain the previously observed photosynthetic increase at elevated CO_2_ and temperature (Eller et al. 2014) as well as the large aboveground biomass production in the elevated climatic conditions observed in the present study.

In contrast to the climatic conditions, soil salinity significantly affected the transcript abundance of all three photosynthetic genes, and twice in interaction with the climatic treatment. The major constraints of salinity on plants are osmotic stress, gas exchange restrictions, ion toxicity, and nutritional imbalance (Greenway and Munns 1980). Plants experiencing osmotic stress due to high soil salinity face a trade-off between decreasing transpiration to minimize water loss and opening their stomata for CO_2_ uptake (Parida and Das 2005). CO_2_ assimilation may thus become limited and the transcription of photosynthesis-related genes may be downregulated. This could be seen in the present study, where the 20‰ salinity at ambient climatic conditions resulted in a lower transcript abundance of the *PRK* and *PGK* genes, whereas the transcript abundance of the *RbcS* gene was hardly affected. In the elevated climatic conditions, however, the expression of *PGK* and *PRK* was not negatively affected by the salt treatment, and the *RbcS* expression was even upregulated. The atmospheric CO_2_ concentration is, however, not the only factor which can affect the assimilation of salt-exposed plants. Lissner et al. (1999) found that temperature also has a significant effect, and showed that reed populations grown in a warmer climate were less affected by salinity than populations from colder areas. Also in our study, the negative salt effect on the aboveground biomass production was ameliorated in the warmer future climate scenario.

*Phragmites australis* tolerates low to intermediate salt concentrations, and some genotypes are even capable of surviving salinity levels as high as 72‰ (Achenbach et al. 2013). By producing osmotically active solutes in its leaves, *P. australis* can improve its osmoregulation and achieve salt exclusion (Lissner and Schierup 1997). However, as this tolerance mechanism includes the investment of photosynthates, it is accomplished only at the expense of biomass production, as seen in the present study.

In the salt-treated plants, particularly under the elevated climatic conditions, the Na^+^/H^+^ antiporter gene, *PhaNHA*, was upregulated, indicating a higher biosynthesis of the antiporter and hence increased Na^+^ exclusion at high salinity. Extrusion of Na^+^ has been shown to be an important ion-detoxification mechanism for *P. australis* (Matoh et al. 1988). As *P. australis* does not have excreting salt glands, Na^+^ is actively exported to the rhizosphere after retranslocation from the leaves to the roots (Matsushita and Matoh 1991). As this mechanism of salt tolerance is energy dependent, factors which affect the plant energy balance, such as photosynthesis, will also affect the resistance to salinity.

Also the expression of the *MnSOD* and *GPX* genes was higher in salt-treated plants, for *GPX* especially at elevated CO_2_ and temperature, confirming our second hypothesis. When high soil salinity reduces CO_2_ uptake, the internal CO_2_/O_2_ ratio decreases and photoreducing capacity accumulates. Oxygen can then be the consequent electron acceptor, generating reactive oxygen species (ROS) that can cause oxidative damage to bio-molecules (Turkan and Demiral 2009). Antioxidative molecules and specific ROS-scavenging enzymes, like those coded for by the *MnSOD* and *GPX* genes, provide protection against oxidative damage (Parida and Das 2005).

The high transcript abundance of *MnSOD* and *GPX* may be an explanation for the ameliorated salt effect under the future climate scenario. This has also been shown in *Aster tripolium*, where the gene expression of different antioxidant enzymes, including SOD and Ascorbate peroxidase, was increased under saline conditions and elevated CO_2_ compared to ambient CO_2_. At the same time, the resulting protein abundances and enzyme activities were increased (Geissler et al. 2009, 2010). Contrary to this, the gene expression of antioxidant enzymes in salt-stressed barley was upregulated at ambient CO_2_ but not at elevated CO_2_ (Perez-Lopez et al. 2009b). Our simulated future climate treatment did not only involve elevated CO_2_ but also higher temperature, which may have contributed to a faster protein turnover and an acceleration of enzymatic activities, reflected in the higher transcription of the genes. Such an energetically costly higher expression and activation of salt-tolerance mechanisms could have been facilitated by a higher photosynthetic activity (Geissler et al. 2010). This, in turn, may have been provided by an increased expression of enzymes involved with photosynthesis, like the *RbcS* gene in the present study.

Plants without salt treatment showed a lower expression of ROS-scavenging enzymes under the elevated compared to the ambient climatic conditions. This probably reflected the diminished need for antioxidative protection in general, as at elevated CO_2_ and higher temperature, ROS formation due to photorespiration is considerably reduced (Leakey et al. 2012).

Although we did not include the North American native *P. australis* ssp. *americanus* in our study, previous research has shown that the native lineage is ecophysiologically inferior to the invasive Eurasian lineage under a range of environmental conditions, including elevated CO_2_ (Vasquez et al. 2005; Mozdzer and Zieman 2010; Mozdzer and Megonigal 2012; Mozdzer et al. 2013). Thus, there is good reason to believe that the two genotypes investigated here and their lineages will remain the more vigorous reeds also under future climate changes at the Gulf Coast of North America.

Overall, the predicted future rise in temperature and atmospheric CO_2_ will facilitate an improvement in salt resistance of the two invasive *P. australis* genotypes investigated here. Sea level rise is a potential threat to the Gulf Coast, and although a higher salt resistance may facilitate invasive vigor of *P. australis*, it may also help the reeds to sustain the marshes in this area. The increased salinity resistance under a future climate scenario can be ascribed to the higher expression of genes for photosynthesis-related and antioxidant enzymes as well as the Na^+^/H^+^ antiporter for salt exclusion. However, the previously observed ecophysiological differences between the two co-occurring invasive genotypes cannot be explained by differences in the transcript abundance of these genes. Our study addressed a climate change scenario with milder, early-season temperatures. As supra-optimal temperatures may occur in the Gulf Coast following climate change, there is also a need for studies that address the effect of heat on the invasive lineages to draw further conclusions on future reed expansion.

## Supporting Information

**Table S1.** *F*-ratios of General Linear Model (GLM) analysis of aboveground biomass and transcript abundance of genes expressed in two invasive genotypes of *Phragmites australis* from the Mississippi River Delta.

**Table S2.** Results for gene expression and aboveground biomass of each of the two invasive genotypes of *P. australis*.
