# Supplementary material for: Expression of major photosynthetic and salt-resistance genes in invasive reed lineages grown under elevated CO2 and temperature
Source: Ecol Evol. 2014 Oct 12;4(21):4161–72. doi: 10.1002/ece3.1282 (PMC4242567; doi:10.1002/ece3.1282)
Supplement: Supplementary file 1 — Table S1. F-ratios of General Linear Model (GLM) analysis of aboveground biomass and transcript abundance of genes expressed in two invasive genotypes of Phragmites australis from the Mississippi River Delta. [file ece30004-4161-SD1.docx]

**Supporting Table S1.** F-ratios of General Linear Model (GLM) analysis of aboveground biomass and transcript abundance of genes expressed in two invasive genotypes of *Phragmites australis* from the Mississippi River Delta. The main factors are “genotype” (Genot; EU-type *vs.* Delta-type), “climatic conditions” (Clim; ambient climatic conditions *vs.* elevated climatic conditions) and “soil salinity” (Sal; 0‰ salinity *vs.* 20‰ salinity). RbcS – Rubisco small subunit; PGK – Phosphoglycerate kinase; PRK – Phosphoribulokinase; PhaNHA – *P. australis* Na^+^/H^+^ antiporter; MnSOD – Manganese Superoxide dismutase; GPX – Glutathione peroxidase; df = degrees of freedom. Statistically significant values are shown in bold: *<0.05 probability level, **<0.01, ***<0.001.

| **Parameter** | **Main factors** | |  |  | **Interactions** | |  |  |
| --- | --- | --- | --- | --- | --- | --- | --- | --- |
|  | *Genot*  *(df = 1)* | *Clim*  *(df = 1)* | *Sal*  *(df = 1)* |  | *Genot × Clim*  *(df = 1)* | *Genot × Sal*  *(df = 1)* | *Clim × Sal*  *(df = 1)* | *Genot × Clim × Sal*  *(df = 1)* |
| *RbcS* | 0.00 | 0.34 | **5.67*** |  | 0.44 | 0.54 | **11.06**** | 0.11 |
| *PGK* | 0.36 | 3.44 | 4.34 |  | 1.94 | 0.17 | 3.97 | 1.18 |
| *PRK* | 1.37 | **4.83*** | **9.77**** |  | 1.73 | 0.77 | **4.55*** | 0.28 |
|  |  |  |  |  |  |  |  |  |
| *PhaNHA* | 0.50 | 0.86 | **6.40*** |  | 1.43 | 0.50 | 1.91 | 0.35 |
| *MnSOD* | 0.34 | 1.06 | **19.03***** |  | 0.14 | 0.24 | 4.08 | 1.83 |
| *GPX* | 1.07 | 0.28 | **18.50***** |  | 0.01 | 0.00 | **38.97***** | 0.04 |
|  |  |  |  |  |  |  |  |  |
| Aboveground biomass | 0.45 | **569.50***** | **408.92***** |  | 2.32 | 0.31 | 0.13 | 1.22 |
