# Supplementary material for: Expression of major photosynthetic and salt-resistance genes in invasive reed lineages grown under elevated CO2 and temperature
Source: Ecol Evol. 2014 Oct 12;4(21):4161–72. doi: 10.1002/ece3.1282 (PMC4242567; doi:10.1002/ece3.1282)
Supplement: Supplementary file 2 — Table S2. Results for gene expression and aboveground biomass of each of the two invasive genotypes of P. australis. [file ece30004-4161-SD2.docx]

**Supporting Table S2:** Total aboveground biomass and relative expression of photosynthesis-related genes of two genotypes belonging to two invasive *Phragmites australis* lineages (EU- and Delta-type) grown under “ambient” and “elevated” climatic conditions, and at 0‰ or 20‰ salinity. Upper and lower limits of 95% Tukey’s HSD intervals are shown for relative gene expression. *RbcS:* Rubisco small subunit; *PGK*: Phosphoglycerate kinase; *PRK*: Phosphoribulokinase; *PhaNHA*: Na^+^/H^+^ antiporter; *MnSOD*: Manganese Superoxide dismutase; *GPX*: Glutathione peroxidase.

| ***Gene*/** | | **Treatment** | | |  | | **Salinity** | |  |  |  |  |
| --- | --- | --- | --- | --- | --- | --- | --- | --- | --- | --- | --- | --- |
| **Parameter** | | | |  | | | 0‰ |  | |  | 20‰ |  |
|  |  | | |  | | | EU | Delta | |  | EU | Delta |
| *RbcS* | Ambient | | | relative expression | | | 100.0 | 101.7 | |  | 91.0 | 97.9 |
|  |  | | | *upper limit* | | | *127.4* | *129.5* | |  | *116.0* | *124.7* |
|  |  | | | *lower limit* | | | *78.5* | *79.8* | |  | *71.5* | *76.9* |
|  |  | | |  | | |  |  | |  |  |  |
|  | Elevated | | | relative expression | | | 88.5 | 78.3 | |  | 122.4 | 125.6 |
|  |  | | | *upper limit* | | | *112.8* | *99.8* | |  | *155.9* | *160.0* |
|  |  | | | *lower limit* | | | *69.5* | *61.5* | |  | *96.1* | *98.6* |
|  |  | | |  | | |  |  | |  |  |  |
| *PGK* | Ambient | | | relative expression | | | 100.0 | 111.3 | |  | 78.0 | 78.7 |
|  |  | | | *upper limit* | | | *128.8* | *143.4* | |  | *100.5* | *101.3* |
|  |  | | | *lower limit* | | | *77.6* | *86.4* | |  | *60.6* | *61.1* |
|  |  | | |  | | |  |  | |  |  |  |
|  | Elevated | | | relative expression | | | 118.7 | 92.0 | |  | 105.7 | 101.9 |
|  |  | | | *upper limit* | | | *152.9* | *118.5* | |  | *136.1* | *131.2* |
|  |  | | | *lower limit* | | | *92.1* | *71.4* | |  | *82.0* | *79.1* |
|  |  | | |  | | |  |  | |  |  |  |
| *PRK* | Ambient | | | relative expression | | | 100.0 | 109.5 | |  | 79.7 | 74.0 |
|  |  | | | *upper limit* | | | *122.7* | *134.4* | |  | *97.7* | *90.7* |
|  |  | | | *lower limit* | | | *81.5* | *89.3* | |  | *65.0* | *60.3* |
|  |  | | |  | | |  |  | |  |  |  |
|  | Elevated | | | relative expression | | | 111.9 | 98.7 | |  | 107.7 | 91.1 |
|  |  | | | *upper limit* | | | *137.2* | *121.1* | |  | *132.2* | *111.8* |
|  |  | | | *lower limit* | | | *91.2* | *80.4* | |  | *87.8* | *74.3* |
|  |  | | |  | | |  |  | |  |  |  |
| *PhaNHA* | Ambient | | | relative expression | | | 100 | 126.7 | |  | 114.7 | 149.8 |
|  |  | | | *upper limit* | | | *158.3* | *200.5* | |  | *181.6* | *237.1* |
|  |  | | | *lower limit* | | | *63.2* | *80.0* | |  | *72.5* | *94.6* |
|  |  | | |  | | |  |  | |  |  |  |
|  | Elevated | | | relative expression | | | 119.3 | 94.2 | |  | 168.7 | 187.8 |
|  |  | | | *upper limit* | | | *188.9* | *149.1* | |  | *267.0* | *297.3* |
|  |  | | | *lower limit* | | | *75.4* | *59.5* | |  | *106.6* | *118.6* |
|  |  | | |  | | |  |  | |  |  |  |
| *MnSOD* | Ambient | | | relative expression | | | 100.0 | 111.1 | |  | 130.8 | 114.7 |
|  |  | | | *upper limit* | | | *124.9* | *138.8* | |  | *163.5* | *143.3* |
|  |  | | | *lower limit* | | | *80.0* | *88.9* | |  | *104.7* | *91.8* |
|  |  | | |  | | |  |  | |  |  |  |
|  | Elevated | | | relative expression | | | 91.9 | 81.7 | |  | 130.9 | 130.1 |
|  |  | | | *upper limit* | | | *114.8* | *102.1* | |  | *163.6* | *162.6* |
|  |  | | | *lower limit* | | | *73.5* | *65.4* | |  | *104.8* | *104.1* |
|  |  | | |  | | |  |  | |  |  |  |
| *GPX* | Ambient | | | relative expression | | | 100.0 | 91.1 | |  | 84.4 | 79.9 |
|  |  | | | *upper limit* | | | *130.9* | *119.3* | |  | *110.5* | *104.5* |
|  |  | | | *lower limit* | | | *76.4* | *69.6* | |  | *64.5* | *61.0* |
|  |  | | |  | | |  |  | |  |  |  |
|  | Elevated | | | relative expression | | | 58.6 | 54.3 | |  | 134.4 | 121.9 |
|  |  | | | *upper limit* | | | *76.7* | *71.1* | |  | *175.9* | *159.5* |
|  |  | | | *lower limit* | | | *44.8* | *41.5* | |  | *102.7* | *93.1* |
|  |  | | |  | | |  |  | |  |  |  |
| Total aboveground biomass | | | Ambient | | | 31.6 ± 3.0 | | 27.6 ± 4.1 | |  | 6.9 ± 0.8 | 7.2 ± 0.7 |
| (g dry mass; n=8; mean ± 1 S.E.) | | | Elevated | | | 144.5 ± 4.6 | | 179.1 ± 16.1 | |  | 35.2 ± 3.1 | 39.8 ± 3.6 |
